# Dilative arteriopathy in Pompe disease may not only affect the cerebral arteries

**DOI:** 10.1016/j.ymgmr.2018.08.003

**Published:** 2018-08-28

**Authors:** Josef Finsterer

**Affiliations:** Krankenanstalt Rudolfstiftung, Messerli Institute, Veterinary University of Vienna, Postfach 20, 1180 Vienna, Austria

**Keywords:** Metabolic myopathy, Cardiomyopathy, Late onset Pompe disease, Arteriopathy, Renal infarction, Intracerebral bleeding

With interest we read the article by Pappa et al. about a 42yo male with late onset Pompe disease (LOPD), initially manifesting with progressive, proximal muscle weakness of the lower limbs and later with dilative arteriopathy of the renal artery [1]. The following comments arose.

We do not agree with the description of the abnormal MRA as fibromuscular dysplasia [1]. Since smooth muscle cells and the autonomic nerves can be affected in LOPD [2], it is conceivable that the abnormality of the right renal artery represents dilative arteriopathy attributable to the underlying metabolic defect, as has been described for the cerebral arteries [3]. Was dilative arteriopathy detected in any other vascular territory?

We also do not agree with the notion that there is a typical gait of LOPD patients [1]. Waddling gait is non-specific and occurs with all myopathies manifesting with weakness of the pelvic girdle or proximal lower limb muscles.

LOPD may also manifest in the myocardium as hypertrophic/dilative cardiomyopathy [4]. Was dyspnoea also attributable to involvement of the myocardium leading to heart failure? Which were the findings on echocardiography and cardiac MRI? Did cardiac MRI show late gadolinium enhancement, an indicator for affection of the myocardium in addition to hypertrophy [5]?

The patient received enzyme-replacement therapy (ERT) and improved within 2y of treatment with regard to the outcome parameter walking distance. Did ERT also exhibit a beneficial effect on dilative arteriopathy?

The patient developed intracerebral bleeding under a double therapy with an antithrombotic agent and oral anticoagulation. Which compounds were actually given?

Overall, the case could be more meaningful if arterial biopsy would have been taken, if the effect of ERT on dilative arteriopathy would have been assessed, if the guidelines for treating renal infarction would be revised, and if the patient would have been thoroughly investigated cardiologically.
